# SNRPD1 confers diagnostic and therapeutic values on breast cancers through cell cycle regulation

**DOI:** 10.1186/s12935-021-01932-w

**Published:** 2021-04-20

**Authors:** Xiaofeng Dai, Lihui Yu, Xiao Chen, Jianying Zhang

**Affiliations:** 1 Wuxi School of Medicine, Jiangnan University, Wuxi, Jiangsu China; 2 School of Biotechnology, Jiangnan University, Wuxi, Jiangsu China; 3 Henan Academy of Medical and Pharmaceutical Sciences, Zhengzhou University, Zhengzhou, Henan China

**Keywords:** *SNRPD1*, Breast cancer, Cell cycle arrest, Prognosis, Therapeutics

## Abstract

**Background:**

*SNRPD1* is a spliceosome-associated protein and has previously been implicated with important roles in cancer development.

**Methods:**

Through analyzing the differential expression patterns and clinical association of splicing associated genes among tumor and tumor adjacent samples across different tumors and among different breast cancer subtypes, we identify the tumor promotive role of SNRPD1 using multiple publicly available datasets. Through pathway, gene ontology enrichment analysis and network construction, we linked the onco-therapeutic role of SNRPD1 with cell cycle. Via a series of experimental studies including knockdown assay, qPCR, western blotting, cell cycle, drug response assay, we confirmed the higher expression of SNPRD1 at both gene and protein expression levels in triple negative breast cancer cells, as well as its roles in promoting cell cycle and chemotherapy response.

**Results:**

Our study revealed that *SNRPD1* over-expression was significantly associated with genes involved in cell cycle, cell mitosis and chromatin replication, and silencing *SNRPD1* in breast cancer cells could lead to halted tumor cell growth and cell cycle arrest at the G_0_/G_1_ stage. We also found that triple negative breast cancer cells with reduced *SNRPD1* expression lost certain sensitivity to doxorubicin whereas luminal cancer cells did not.

**Conclusions:**

Our results suggested the prognostic value of *SNRPD1* on breast cancer survival, its potential as the therapeutic target halting cell cycle progression for breast cancer control, and warranted special attention on the combined use of doxorubicin and drugs targeting *SNRPD1*.

**Supplementary Information:**

The online version contains supplementary material available at 10.1186/s12935-021-01932-w.

## Introduction

**Fig. 1:**
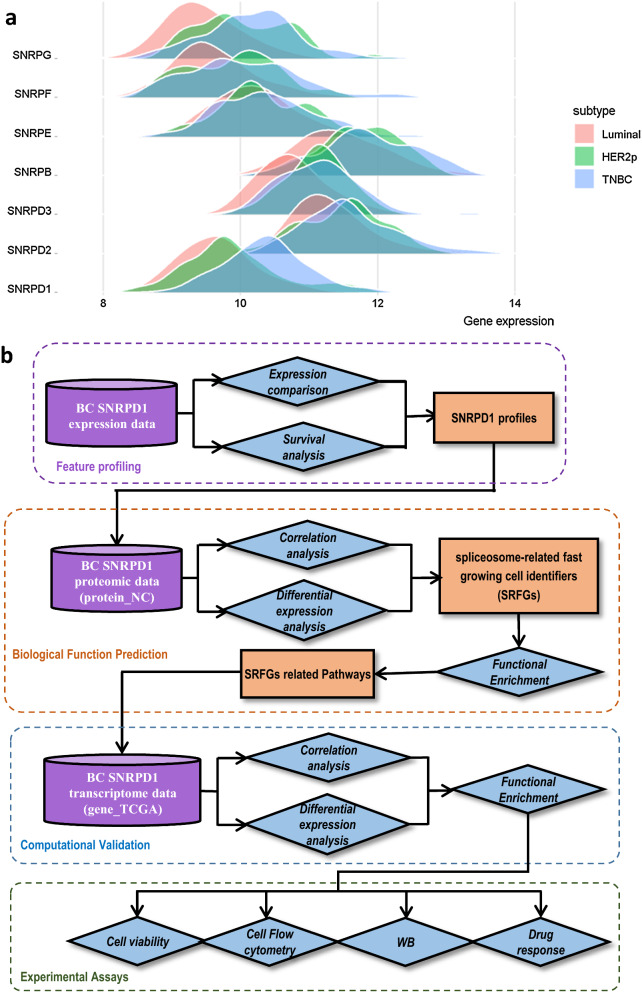
Rational and workflow of this study. **a** Distribution of the gene expression of spliceosomal core Sm proteins across breast cancer subtypes using TCGA transcriptomic data (gene_TCGA). **b** Study workflow

Spliceosome is a dynamic complex that catalyzes the splicing of precursor RNA into mRNA in eukaryotic cells and comprised of 5 small nuclear ribonucleoproteins (snRNPs), i.e., U1, U2, U4, U5, U6, and more than 200 polypeptides [1, 2]. Most spliceosomal snRNPs contain a common set of core Sm proteins, i.e., SNRPB, SNRPD1, SNRPD2, SNRPD3, SNRPE, SNRPF, SNRPG [2]. It is widely acknowledged that accurate splicing is essential to ensure normal cell functionalities such as cell cycle, apoptosis, migration and invasion [3–8]. It was observed that altered expression of genes involved in the splicing machinery was correlated with the incidence of hematological diseases such as chronic lymphocytic leukemia and myelodysplasia [9–12]. We analyzed the transcriptomic profiles of the 7 core Sm proteins across 31 cancer types and among breast cancer subtypes, and found that SNRPD1 had the highest number of cancers with over 2 folds up-regulation between cancer and normal tissues (Table 1), and the distribution of *SNPRD1* could be nicely split into two sub-distributions by TNBC and non-TNBC while the other genes did not (Fig. 1a). In addition, spliceosome assembly components were revealed as the most enriched pathway deregulated in breast cancers with SNRPD1 being an important player according to exonic expression profiling of 120 breast tumors and 45 benign lesions [13]. A recent reprint identified SNRPD1 as one of the top 10 essential ribosome binding proteins for breast cancer survival from both the genome-scale RNAi loss-of-function screens (DEMETER2) and the genome-scale CRISPR-Cas9 loss-of-function screens (CERES) [14]. SNRPD1 over-expression was used to define subsets of highly aggressive cancers [15] and was proposed as therapeutic targets of multiple cancers such as melanoma, lung and breast tumor cells as a result of induced autophagy [15].

**Table 1:**
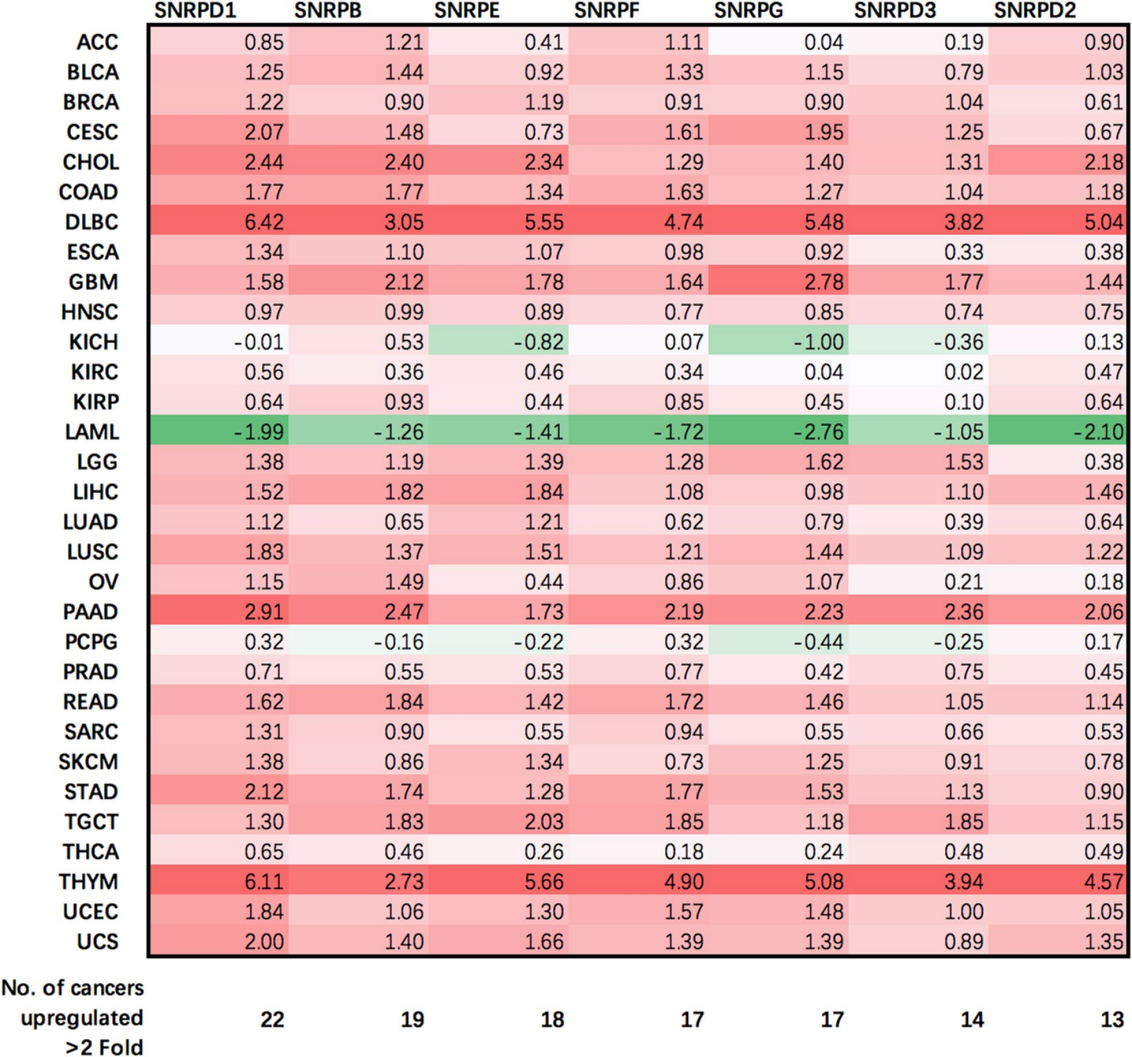
Log_2_ fold change of gene expression between tumor and adjacent normal tissues across 31 cancer types using transcriptomic data stored in TCGA

We therefore decided to focus on SNRPD1 and explore its potential diagnostic and therapeutic values in breast cancers.

Through computational predictions followed by experimental validations, we identified from this study that *SNRPD1* over-expression is prognostic of high malignancies among breast cancer patients due to accelerated cell cycle, and proposed that targeting SNRPD1 could halt cell cycle progression at the G_0_/G_1_ phase for effective breast cancer management.

## Materials and methods

### Computational analysis

#### Data

The breast cancer quantitative proteomic data set, which is comprised of 9995 proteins and 45 samples, was downloaded from https://www.nature.com/articles/s41467-019-09018-y [16], namely the ‘Protein_ NC’ dataset.

TCGA breast cancer transcriptomic data together with the clinical data were retrieved from TCGA [17] data portal (http://cancergenome.nih.gov) on Date April 1st 2018. Files containing patients’ transcriptome data were combined into a single matrix comprised of 20,531 genes and 931 patients including 84 death events. The maximum follow-up time on overall survival (OS) is 120 months. This dataset contains 110 TNBC patients, 36 HER2 positive patients, and 785 luminal patients. The mRNA data was log2 transformed before data processing. This data was referred to as the ‘gene_TCGA’ dataset in this study.

Gene expression data of splicosome core proteins across 31 cancers were retrieved from GEPIA2 [18], which holds RNA sequencing data of 9736 tumors and 8587 normal samples from the TCGA and the GTEx (Genotype-Tisuse Expression) projects.

Breast cancer cell line microarray data consisting of 183 primary breast tumor samples was downloaded from https://www.ebi.ac.uk/arrayexpress/experiments/E-MTAB-181/ [19, 20]. The data was quantile-normalized followed by log2 transformation, and referred to as the ‘gene_CLM’ dataset in this study.

The web interface tool named ‘GEPIA’ was used to retrieve TCGA mRNA expression data of input genes across all cancer types [18].

#### Survival analysis

The 10-year breast cancer OS analysis and relapse free survival (RFS) analysis of SNRPD1 were performed using Kaplan Meier plotter [21] (http://kmplot.com/analysis/). A p-value < 0.05 from the log rank test was used as the threshold to assess the test statistical significance.

#### Enrichment analysis

Gene Ontology (GO) [22] and Kyoto Encyclopedia of Genes and Genomes database (KEGG) [23] enrichment analysis were performed using the R package ‘clusterProfiler’ and ‘org.Hs.db.eg’ [24]. Fisher's exact test was utilized to measure the significance of GO terms and biological pathways. The p-values were adjusted using Benjamini–Hochberg false discovery rate (FDR), and p < 0.01 was considered as the significance threshold [25]. Gene Set Enrichment Analysis (GSEA) was performed to test each functional biological term.

#### Hierarchical clustering

Samples and genes were clustered in a form of heatmap using the ‘pheatmap’ function, which uses the hierarchical clustering function ‘hclust’ with ‘distance’ as the correlation and ‘ward.D2′ as the clustering method. Patient subtype was annotated using different colors on the top of the heatmap.

#### Correlation analysis

Pearson correlation was calculated using the ‘gene_TCGA’ dataset to evaluate the correlations of SNRPD1 with Ki67, ER, and HER2. The calculation was performed in R using function ‘corr’.

#### Correlation analysis

Receiver operating characteristic (ROC) curves were calculated using the R package ‘ROCR’ to compare the performances of SNRPD1 and KI67 in prognosing triple negative breast cancers.

### Experiments

#### Cell culture

The luminal cell line MCF7 and TNBC cell line MDAMB231 were used. Both cells were stored in liquid nitrogen in 90% FBS and 10% DMSO solution, thawed in DMEM medium supplemented with 10% fetal bovine serum (Gibco), and cultured at 37 °C with 5% CO_2_.

#### siRNA design

Two siRNAs targeting *SNRPD1* were synthesized by company GenePharma (Additional file 1: Table S1) and pooled together on usage. GenePharma Silencer Select Negative Control was used as the negative control.

#### Q-PCR

Total RNA was extracted using TRIzol reagent (TianGen) 24 h after siRNA transfection, following reverse transcription into cDNA using PrimeScriptRT reverse transcriptase (Takara). Primers for Q-PCR were listed in Additional file 1: Table S2. The qPCR experiments were conducted using the qPCR kit (CWbio) following the manufacture’s protocol and using the Roche LightCycler 480 qPCR system. The relative expression levels were calculated using the 2^−△△Ct^ methods. Student T test was used to evaluate the statistical significance with p value < 0.05 being considered statistically significant. Primers designed for qPCR were listed in Additional file 1: Table S2.

#### Western blot

Total protein was extracted 48 h after transfection using RIPA lysis buffer supplemented with protease inhibitors. The protein concentration was estimated using the BCA Protein Assay Kit (Tiangen) following the standard protocol. Protein samples were separated on SDS polyacrylamide gel and transferred to polyvinylidene difluoride (PVDF) membranes using BioRad wet transfer apparatus. The membrane was incubated with primary antibodies overnight at 4℃ and with secondary antibodies for 2 h after being blocked in 5% non-fat milk for 1 h at the room temperature. The signal was detected using Tanon High-sigECL western Blotting substrate reagents and BioRad imaging apparatus.

#### Proliferation assay

Cells were prepared in 96-well plates with ~ 50% confluency before transfection. The siRNAs and lipo3000 reagents were mixed in Opti-MEM medium for 15–20 min before transfection with a final siRNA concentration being 20 nl per well. Cell proliferation was measured using CKK-8 (Dojindo) 48 h after transfection, and luminescence was detected using EZ Read 800 microplate Reader (Biochrom) after incubation at 37ºC for 2 h. Student T test was performed using R to evaluate cell viability reduction with p value < 0.05 being considered statistically significant.

#### Cell flow cytometry

Cell flow cytometry was performed 48 h after siRNA transfection. Cells were collected using EDTA-free trypsin, washed twice using 0.5 ml PBS, suspended in cold 70% ethanol, and stored in 4 °C overnight. Ethanol was removed and cells were re-suspended in PBS the next day followed by 0.05 mg/ml Propidium Iodide (PI) addition. Cells were kept in darkness on ice for 30 min before being sent to BD C6 flow cytometry. Analysis was performed using the flowjo software.

#### Doxorubicin resistance assays

Various concentrations of doxorubicin (1 nM, 10 nM, 100 nM, 1000 nM, 10,000 nM) with 3 replicates were used for both control and *SNRPD1* knocked down cell-lines. Doxorubicin (Sigma) was added 24 h after siRNA transfection. 10ul per well of CKK-8 regent was added 96 h after transfection, and luminescence was detected using EZ Read 800 microplate Reader after cell incubation at 37ºC for 2 h. The dose–response curve and half-maximum inhibitory concentration (IC50) values were obtained using the ‘drc’ package [26] in R, where a four parameter log-logistic model (LL.4) was used for data fitting. Statistical significance on IC50 alteration was evaluated by student T test.

The workflow of this study was presented as Fig. 1b.

## Results

### Bioinformatics analysis predicts the association of SNPRD1 with cell cycle

**Fig. 2:**
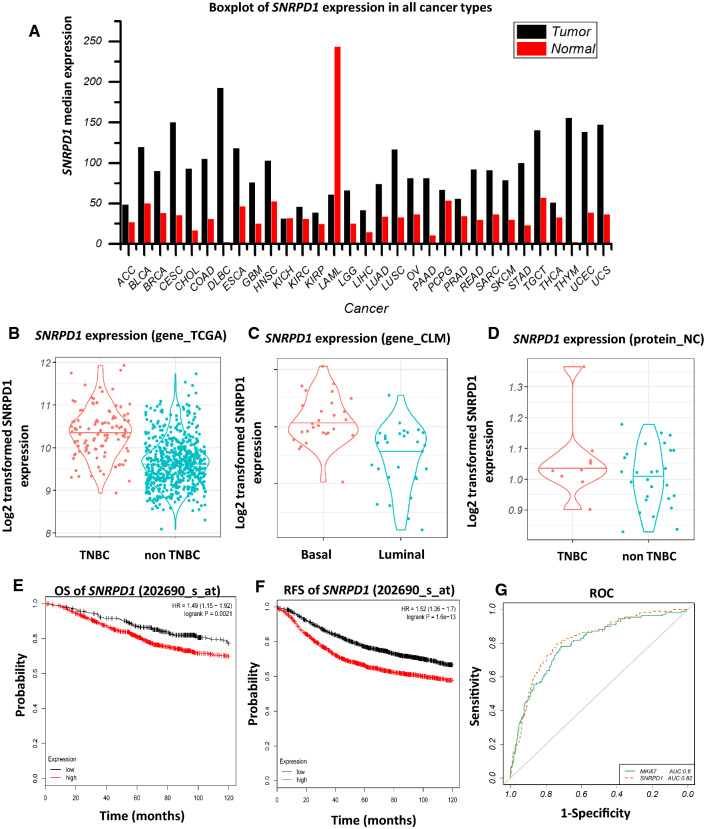
Expression profiles and clinical relevance of *SNRPD1*. **a** *SNRPD1* expression across all cancer types in the gene_TCGA dataset. Expansion of abbreviations of the tumor names is listed in Additional file 1: Table S5. *SNRPD1* expression across breast cancer subtypes in **b** gene_TCGA, **c** gene_CLM, and **d** protein_NC datasets. Overall survival (**e**) and relapse free survival (**f**) of *SNRPD1* in breast cancer patients drawn using Kaplan Meier Plotter. **g** ROC curves of SNRPD1 and KI67

*SNRPD1* expression is higher in malignant or highly proliferative cells than normal cells in all types of cancers except for LAML (Acute Myeloid Leukemia) according to TCGA mRNA data (Fig. 2a). Triple negative breast cancers (TNBCs) are more malignant and grow faster than the other breast cancer subtypes [27], which exhibited higher *SNRPD1* expression than non-TNBCs in TCGA patient transcriptomic data (p = 8.4E−4, Fig. 2b) and patient protein data protein_NC (p = 0.0016, Fig. 2d). Basal breast cancer cells are the counterpart of TNBCs at the cell line level, which showed higher *SNRPD1* expression than non-basal cells according to the CLM cell line gene expression data (p < 2E−16, Fig. 2c). Both OS and RFS analyses showed that high *SNRPD1* expression was prognostic of unfavourable clinical outcome with statistical significance (HR = 1.49, p = 0.0021 for OS, HR = 1.52, p = 1.6E−13 for RFS**,** Fig. 2e, f). ROC curves showed the performances of SNRPD1 and KI67 in prognosing triple negative breast cancers (AUC = 0.82 for SNRPD1, AUC = 0.8 for KI67, Fig. 2g).

We defined genes differentially expressed between TNBC and non-TNBC cells and highly correlated with *SNRPD1* expression as ‘spliceosome-related fast-growing cell identifiers’ (SRFGs) and identified 434 SRFGs from the proteomic data (Additional file 1: Table S3).

**Fig. 3:**
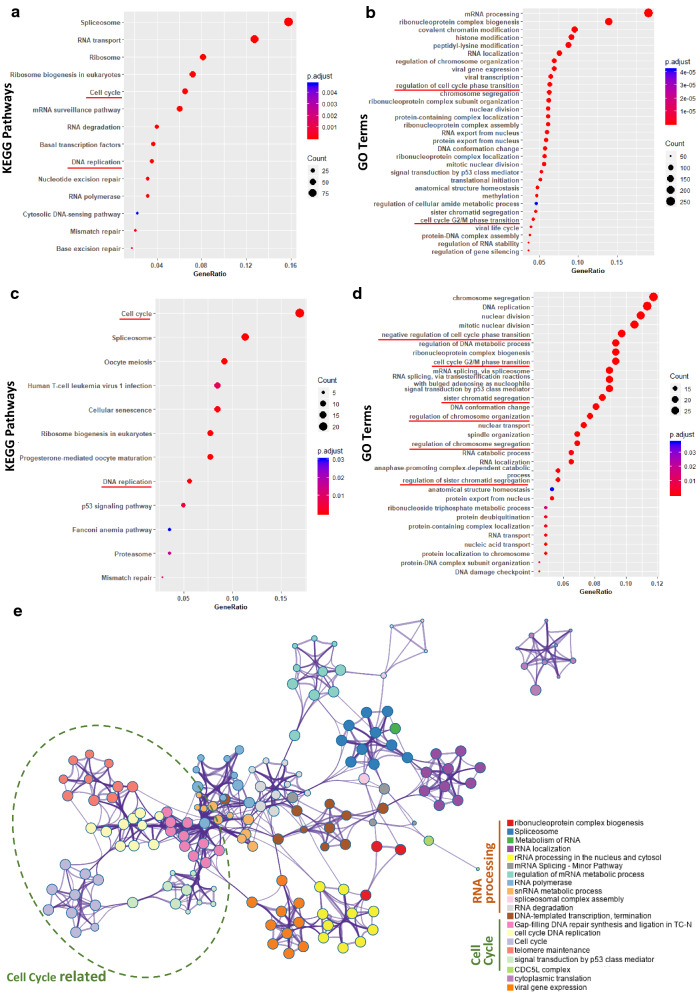
Functional enrichment of SRFGs. **a** KEGG and **b** GO enrichment of SRFGs using the protein_NC dataset. **c** KEGG and **d** GO enrichment of SRFGs using the gene_TCGA dataset. **e** Network of enriched GO terms constructed using protein_NC data

GO and KEGG pathway enrichment analyses showed that SRFGs were enriched in ‘cell cycle’, ‘DNA replication’ and ‘mitosis’ using both the ‘protein_NC’ proteomic (Fig. 3a, b) and ‘gene_TCGA’ transcriptomic data (Fig. 3c, d). It was shown that ‘DNA transcription’, ‘DNA repair’ and ‘Cell cycle’ were the most significantly enriched GO terms besides ‘splicing’. A network depicting the relationship between the enriched GO terms was constructed using SRFGs from the protein_NC data, where each node represents an enriched GO term and nodes with similarities > 0.3 were connected by edges. The nodes in the GO term network were categorized according to their general functionalities, where ‘RNA processing’ and ‘cell cycle’ were popped up as two major clusters. The ‘cell cycle’ cluster was primarily comprised of 5 inter-connected sub-clusters (Fig. 3e).

**Fig. 4:**
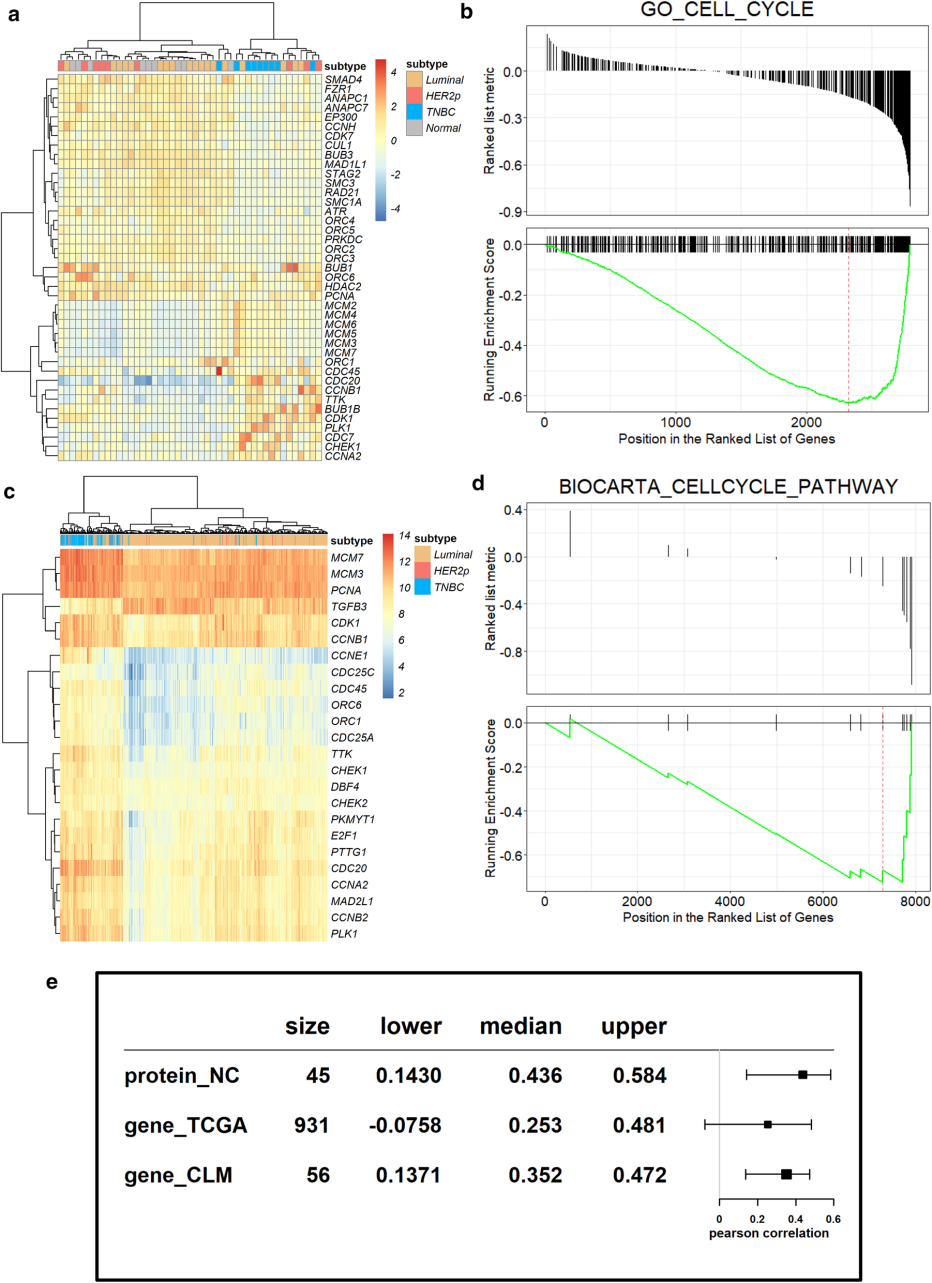
Assessment of the relationship between SNRPD1 and cell cycle. **a** Heatmap drawn using cell cycle related genes from SRFGs, and **b** GSEA of ‘GO_CELL_CYCLE’ using the protein_NC dataset. **c** Heatmap drawn using cell cycle related genes from SRFGs, and **d** GSEA of ‘BIOCARTA_CELLCYCLE_PATHWAY’ using the gene_TCGA dataset. **e** Forest plot showing correlations between SNRPD1 and cell cycle related genes from SRFGs across multiple datasets

In order to confirm the role of cell cycle in breast cancer development, unsupervised hierarchical clustering of protein and mRNA data was performed. TNBC patients were clustered together using cell cycle genes from SRFGs as the classifier using both the proteomic data (Fig. 4a) and the transcriptomic data (Fig. 4c), suggestive of the important role of cell cycle in differentiating TNBC and non-TNBC patients (Fig. 4a, c). GSEA further confirmed the enrichment of cell cycle related genes in SRFGs using both protein_NC and gene_TCGA datasets (Fig. 4b, d).

Correlation analysis showed that *SNPRD1* expression was highly correlated with cell cycle, with the correlation scores being 0.44, 0.25 and 0.35, respectively, in the protein_NC, gene_TCGA and gene_CLM datasets.

### Experimental validation confirms the role of SNRPD1 in cell cycle control

**Fig. 5:**
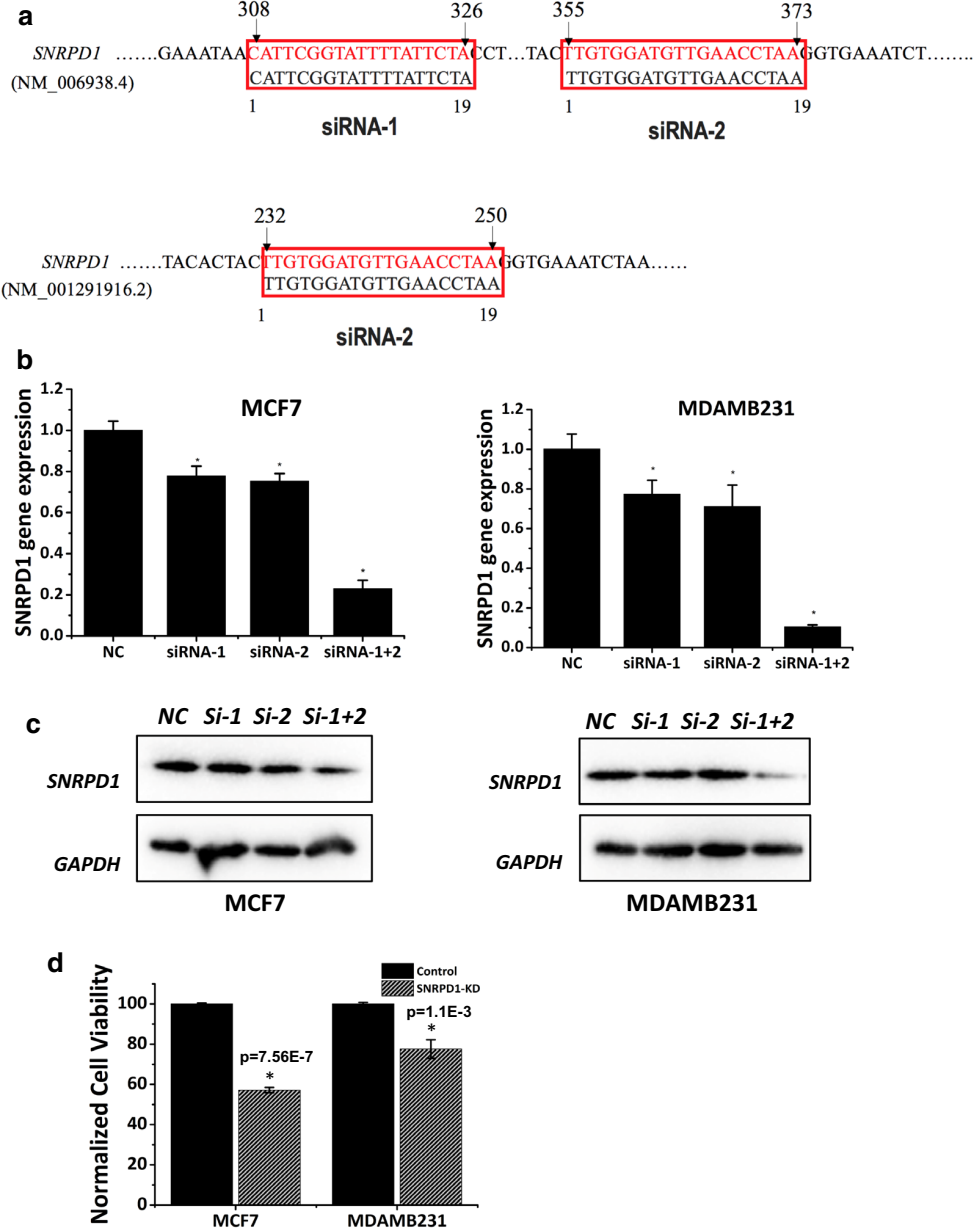
Cell viability upon knocking down *SNRPD1*. **a** Sequence alignment results showing the target loci of the two siRNAs on *SNRPD1*. Knocking down efficiency of siRNAs used for silencing *SNRPD1* in MCF7 and MDAMB231 cells as assessed using **b** q-PCR and **c** western blot. ‘si-1’, ‘si-2’ and ‘si-1 + 2’ each represents siRNA-1, siRNA-2 and their pooled effects. **d** Normalized cell viability upon *SNRPD1* silencing using pooled siRNAs

Two siRNAs were designed (Fig. 5a) and purchased (Additional file 1: Table S1). E value was used to assess the significance of the homologous similarity of two sequences, where two sequences with E < 10E−5 were considered highly homologous and such a homology was nearly confirmed without a need of further validation if E < 10E−6. The siRNA-1 could target the NM_006938.4 transcript and the siRNA-2 could target both the NM_006938.4 and the NM_001291916.2 transcripts. None of these two siRNAs could target cell cycle related siRNAs assessed in this study with statistically significant E value (Table 2).

**Table 2:** Sequence alignment of two designed *SNPRD1* siRNA against *SNRPD1* and cell cycle related genes experimentally assessed in this study

Type	Gene	Accession	E value	Significance
siRNA-1	PCNA	NM_182649.2	0.18	
	CCND1	NM_053056.3	2.4	
	CCNB1	NM_031966.4	0.29	
	CDK1	NM_001786.5	0.068	
	CDCA5	NM_080668.4	1.4	
	NDC80	NM_006101.3	0.077	
	CCNA2	NM_001237.5	0.39	
	**SNRPD1**	**NM_006938.4**	**0.0000002**	*
	SNRPD1	NM_001291916.2	0.16	
siRNA-2	PCNA	NM_182649.2	0.012	
	CCND1	NM_053056.3	0.15	
	CCNB1	NM_031966.4	0.29	
	CDK1	NM_001786.5	/	
	CDCA5	NM_080668.4	1.4	
	NDC80	NM_006101.3	0.3	
	CCNA2	NM_001237.5	0.39	
	**SNRPD1**	**NM_006938.4**	**0.0000002**	*
	**SNRPD1**	**NM_001291916.2**	**0.0000002**	*

Both siRNAs could significantly silence *SNRPD1* (p = 0.002 for siRNA-1 and p = 8.6E−4 for siRNA2 in MCF7; p = 0.0091 for siRNA-1 and p = 0.0093 for siRNA2 in MDAMB231), and we obtained considerably improved inhibitory effects on SNRPD1 expression by pooling these two siRNAs together (p = 1.22E−5 in MCF7, p = 1.64E−5 in MDAMB231, Fig. 5b). Similarly, *SNRPD1* was effectively knocked down in MCF7 and MDAMB231 cells at the protein expression level (Fig. 5c). We therefore used pooled siRNAs in the following assays. In particular, *SNRPD1* mRNA expression was reduced to less than 10–25% of that of the control cells at the gene expression level (Fig. 5b), and about 5–11% of that of the control at the protein expression level (Fig. 5c) upon pooled siRNA transfection.

**Fig. 6:**
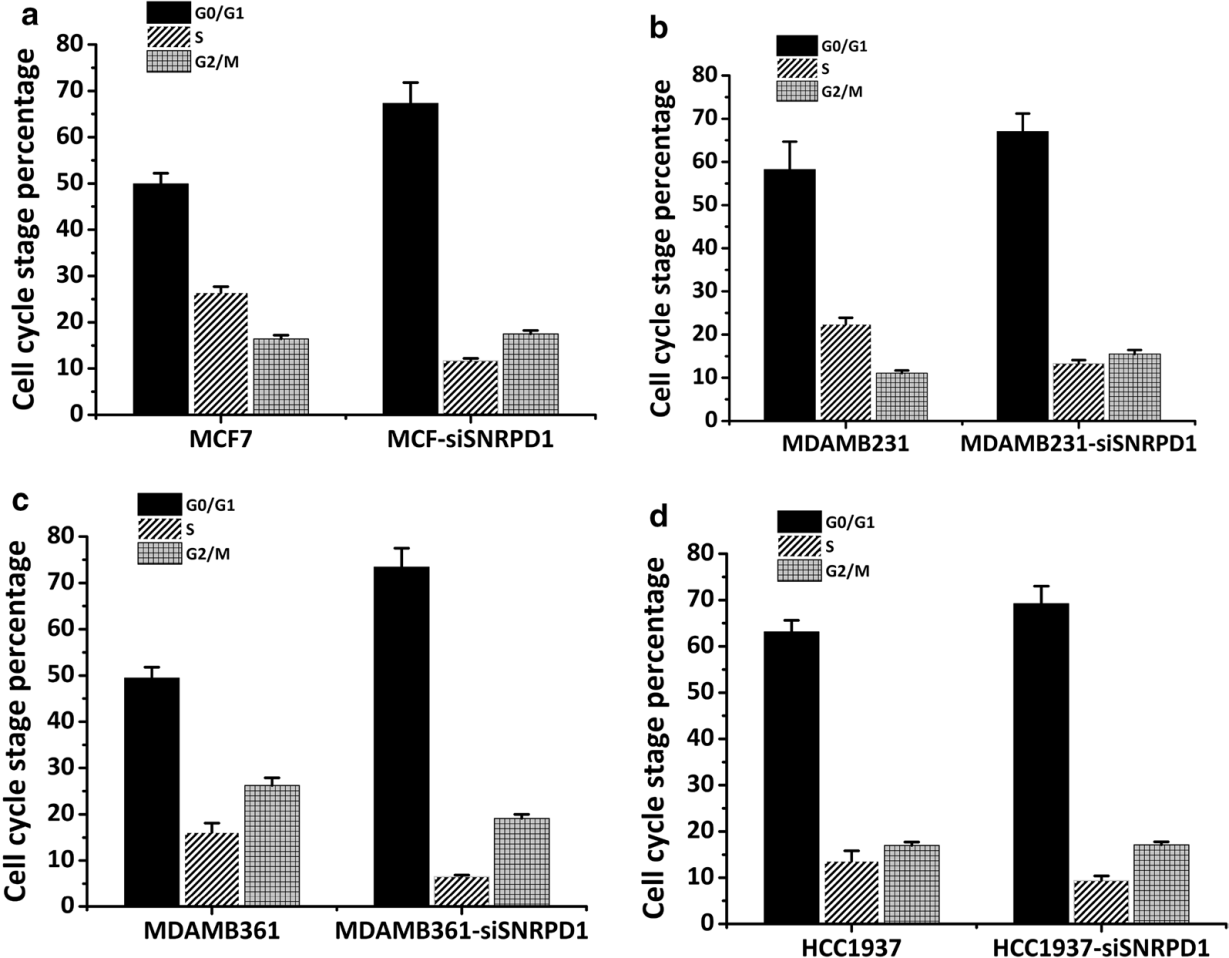
Cell cycle alteration measurement upon knocking down *SNRPD1*. Quantified Cell cycle profiles as measured by cell flowmetry upon *SNRPD1* silencing in **a** MCF7 cell line, **b** MDAMB231 cell line, **c** MDAMB361 cell line, **d** HCC1937 cell line

Both MCF7 and MDAMB231 cells were subjected to reduced cell viability on *SNRPD1* knockdown (p = 7.56E−7 for MCF7, p = 1.1E−03 for MDAMD231, Fig. 5d). Significant discrepancies in G1/G2 proportion were observed between the control and si-*SNPRD1* cells, suggestive of the important role of *SNRPD1* in ‘cell cycle’. Cells were arrested at the G1 phase, resulting in 35% increase of G1 phase cells and 55.7% decrease of S phase cells, and a slight increase of G2 phase cells were observed in si-*SNRPD1* cells (Fig. 6a, b). Similar results were observed in MDAMB361 and HCC1937 cell lines (Fig. 6c, d).

**Fig. 7:**
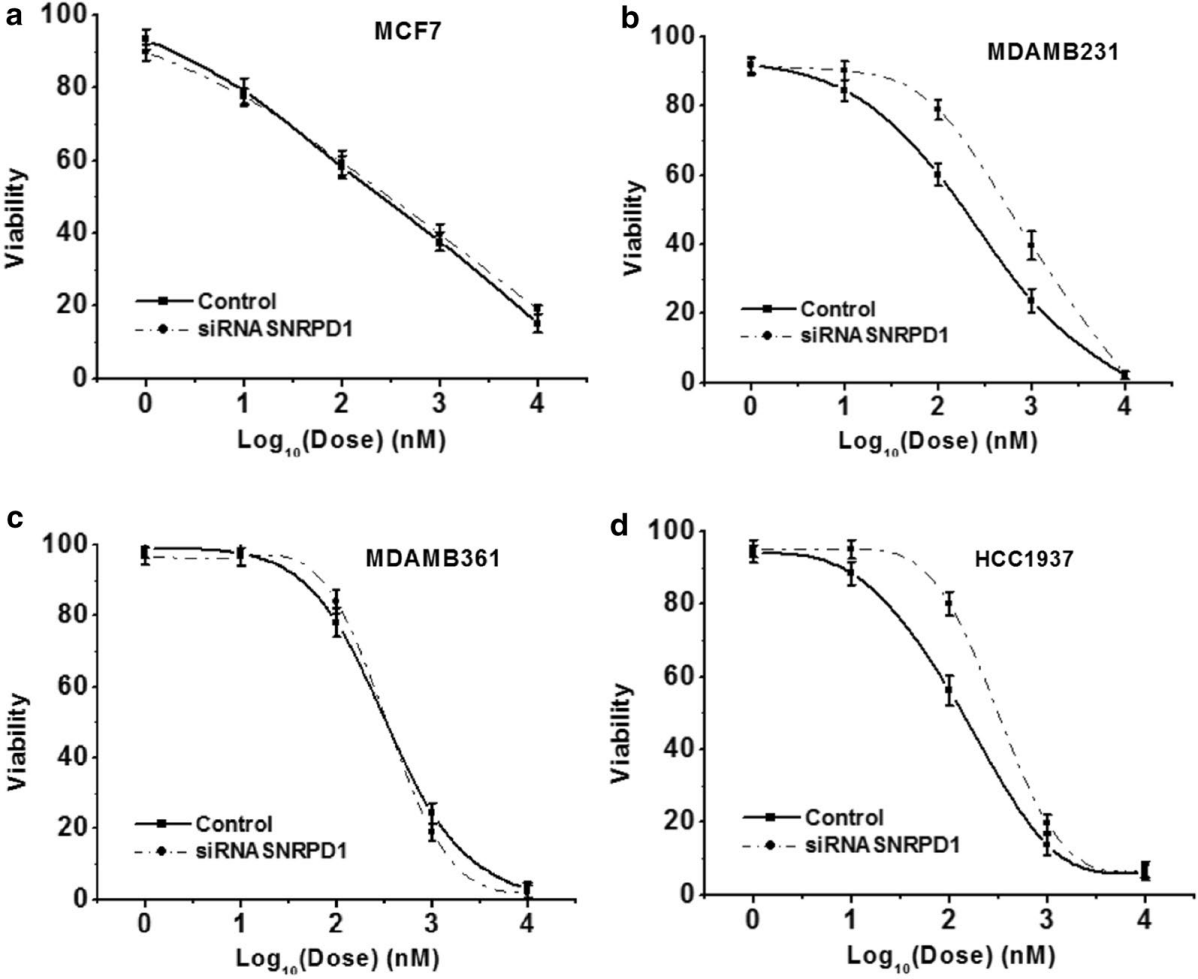
Cell viability in response to Doxorubicin upon knocking down *SNRPD1*. Drug response curves with and without silencing *SNRPD1* in **a** MCF7 cell line, **b** MDAMB231 cell line, **c** MDAMB361 cell line, **d** HCC1937 cell line

Doxorubicin is one type of anthracycline-like drugs that confers cytotoxicity through its antimitotic activity and thus is effective in killing cells with accelerated cell cycle progression including malignant cells. By applying doxorubicin to *SNRPD1-*silenced cells, we observed significantly right-ward shifted IC50 in triple negative breast cancer cells MDAMB231 and HCC1937 (Fig. 7b, 7d) but not in luminal cells MCF7 and MDAMB361 (Fig. 7a, c).

**Fig. 8:**
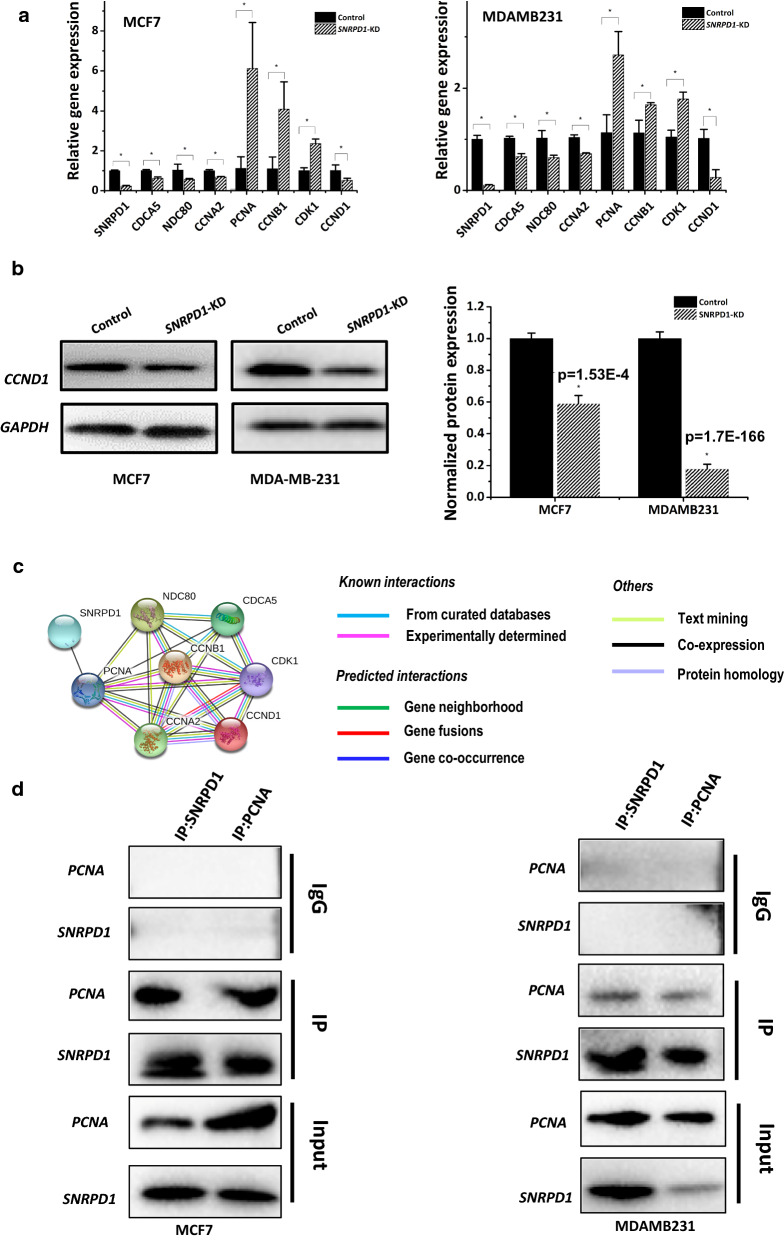
Expression of cell cycle related genes and potential mechanism of *SNRPD1* in cell cycle regulation. **a** Expression of cell cycle related genes *CDCA5, NDC80, CCNA2, PCNA, CCNB1, CDK1, CCND1* after silencing *SNRPD1* at the mRNA level in MCF7 and MDAMB231 cells. **b** Expression of cell cycle related gene *CCND1* after silencing *SNRPD1* at the (**b**) proteomic levels together with its quantified signal intensity in MCF7 and MDAMB231 cells. **c** Predicted protein–protein interaction network of SNRPD1 and cell cycle related proteins using STRING. **d** Immunoprecipitation of SNRPD1 and PCNA in MCF7 and MDAMB231 cells

There were 92 SRFGs enriched in the cell cycle pathway (HSA-1640170, Additional file 1: Table S4) as predicted using STRING [28]. By classifying these genes into four categories, i.e., ‘M’ (genes specific to M phase regulation), ‘M checkpoint’ (genes specific to M phase checkpoint regulation), ‘S’ (genes specific to S phase regulation), ‘S checkpoint’ (genes specific to S phase checkpoint regulation), we identified *CDCA5* as the sole gene specific to M and S phase regulation, 30 and 12 genes specific to M and S phase checkpoint regulation, respectively (Table 3). We chose one gene from each of the four categories, i.e., *CDCA5* (represents both ‘M’ and ‘S’)*, NDC80, CCNA2*, three genes from G1/S transition (*CCNB1*, *CDK1*, *PCNA*, Table 4) to examine whether these cell cycle related genes could be significantly modulated by *SNRPD1* silencing in vitro*.* All tested genes were significantly altered on *SNRPD1* silencing in both MCF7 (p = 8.2E−4 for *CDCA5*, p = 0.027 for *NDC80*, p = 6.2E−4 for *CCNA2*, p = 0.013 for *CCNB1*, p = 5.1E−4 for *CDK1*, p = 0.011 for *PCNA*, Fig. 8a) and MDAMB231 cells (p = 3.56E−4 for *CDCA5*, p = 0.0062 for *NDC80*, p = 2.96E−4 for *CCNA2*, p = 0.009 for *CCNB1*, p = 0.001 for *CDK1*, p = 0.005 for *PCNA*, Fig. 8a).

**Table 3:** Classification of genes enriched in the cell cycle from Reactome pathways among the 434 SRTNS

M		M checkpoint		S	S checkpoint	Rest	Common
AAAS	NCAPG2	BUB1B	**NDC80**	**CDCA5**	**CCNA2**	BLM	PSMD3
**CDCA5**	NCAPH	CCNB1	NUF2	CUL1	CDC45	CDC7	PSMD14
CEP152	NCAPH2	CDC20	NUP107	FEN1	MCM2	CHEK1	RPS27A
HAUS1	NDC1	CDCA8	NUP133	PCNA	MCM3	GMNN	
HAUS2	NUP153	CDK1	NUP160	POLA1	MCM4	MCM10	
HAUS3	NUP155	CENPF	NUP85	POLA2	MCM5	MDC1	
HAUS4	NUP205	CENPH	PLK1	POLE	MCM6	MND1	
HAUS5	NUP210	CENPI	RCC2	PRIM1	MCM7	TOP3A	
HAUS6	NUP50	CENPO	SKA1	PRIM2	ORC6	TOPBP1	
HAUS8	NUP93	CENPQ	SPC24		RFC2	TPX2	
KIF20A	SMC2	CENPU	SPC25		RFC4	WHSC1	
MASTL	SMC4	ERCC6L	XPO1		RFC5		
NCAPD2	VRK1	INCENP	ZW10				
NCAPD3		KIF2C	ZWILCH				
NCAPG		KNTC1	ZWINT				

STRING version 11.0 was used to conduct the enrichment analysis. Experimentally tested genes are highlighted in bold face. ‘M’ and ‘S’ each represents genes specific the M and S phase, respectively. ‘M checkpoint’ and ‘S checkpoint’ each means genes specific to M and S phase check point regulation, respectively, which were obtained by taking the intersection of genes between ‘M phase’ or ‘S phase’ and ‘cell cycle checkpoint’ pathways. ‘Common’ and ‘Rest’ each represents genes present in and absent from all ‘M’, ‘M checkpoint’, ‘S’, ‘S checkpoint’ categories, respectively

**Table 4:** Classification of genes enriched in cell cycle transitions from Reactome pathways among the 434 SRTNS

G1/S transition		G2/M transition	
**CCNA2**	MCM6	CCNA2	HAUS8
**CCNB1**	MCM7	CCNB1	PLK1
CDC45	ORC6	CDK1	PSMD14
CDC7	**PCNA**	CENPF	PSMD3
**CDK1**	POLA1	CEP152	RPS27A
CUL1	POLA2	CUL1	TPX2
GMNN	POLE	HAUS1	XPO1
MCM10	PRIM1	HAUS2	
MCM2	PRIM2	HAUS3	
MCM3	PSMD14	HAUS4	
MCM4	PSMD3	HAUS5	
MCM5	RPS27A	HAUS6	

STRING was used to conduct the enrichment analysis. Experimentally tested genes involved in G1/S transition are highlighted in bold face

We, in addition, tested the expression of CCND1 whose down-regulation is associated with G_0_/G_1_ arrest [29] but missed from the dataset we used for SRFG identification. *CCND1* was down-regulated to approximately 20% and 60% of the control in *SNPRD1*-silenced MCF7 and MDAMB231 cells at both gene and protein expression levels (p = 0.022 at the gene expression level, p = 1.53E−4 at the protein expression level in MCF7, p = 0.0023 at the gene expression level, p = 1.7E−166 at the protein expression level in MDAMB231, Fig. 8b), suggestive of a G_0_/G_1_ cell cycle arrest.

We next explored whether SNRPD1 directly interacts with cell cycle related genes. By constructing a protein–protein interaction network of SNRPD1 and the analyzed cell cycle proteins using STRING version 11.0 (https://string-db.org), we found that SNRPD1 is co-expressed with PCNA with a potential direct interaction (Fig. 8c). We thus conducted immunoprecipitation to assess the interactions of SNPRD1 with PCNA in MCF7 and MDAMB231 cells, and the results showed that SNPRD1 physically interacts with PCNA in both cell lines (Fig. 8d).

## Discussion

Through computational predictions followed by experimental validations, we proposed in this study that *SNRPD1* over-expression contributes to cell cycle progression whose differential expression is prognostic of breast cancer outcome and associated with breast cancer subtypes, and targeting SNRPD1 could lead to cell cycle arrest at the G_0_/G_1_ stage.

Downward-regulated expression of *CDCA5 *[30, 31], *NDC80 *[32], *CCNA2* [33] on *SNRPD1* silencing suggested reduced synthesis of DNA and proteins as well as recessed cell mitosis. Further evidence on reduced CCND1 expression after knocking down *SNRPD1* implicated the accumulation of cells in the G_0_/G_1_ state given the regulatory role of CCND1 in triggering G_0_/G_1_ cell cycle arrest [29].

Results from cell flow cytometry showed that reduced *SNRPD1* could lead to considerable reduction on the S phase, supporting the hypothesis that cells are arrested at the G_0_/G_1_ stage and explaining the mechanism leading to reduced cell viability and increased Anthracycline resistance on knocking down *SNRPD1*.

It was worth noticing that the role of *SNPRD1* in cell cycle arrest is independent of breast cancer subtype though cell cycle related genes could effectively cluster breast cancers into distinct clinically relevant subtypes. This is because that although cell cycle progression is deterministic of cell proliferation, migration and consequently associated with cancer aggressiveness and subtyping, halting cells at a certain cell cycle stage could unanimously block cells from progression regardless of the types of tumor cells.

Being a spliceosomal core Sm protein, SNRPD1 could nicely stratify breast cancer subtypes into TNBCs and non-TNBCs (Fig. 1a), showed comparable sensitivity and accuracy with the canonical proliferation marker KI67 in breast cancer subtyping (Fig. 2g), and exhibited similar correlations with KI67 (cor = 0.38, p = 9.2E−33) and the primary breast cancer subtyping marker ER (cor = − 0.39, p = 4.1E−36), suggesting the prognostic value of SNRPD1 on breast cancer subtyping and its relevance with cell proliferation.

As doxorubicin is a chemotherapy that is known to target highly proliferative cells and typically used to treat TNBCs (TNBC cells are more sensitive to doxorubicin treatment and have faster cell cycle progression due to their more aggressive nature as compared with luminal cancer cells) in clinics, silencing *SNRPD1* would dampen its anti-cancer efficacy if *SNRPD1* over-expression was associated with enhanced cell cycle progression. As expected, doxorubicin resistance in response to *SNRPD1* silencing was observed in triple negative cell lines but not luminal cells, which complies with our findings on the role of *SNRPD1* in cell cycle. Our results also warrant special attention in the combined use of drugs targeting *SNRPD1* and anthracycline-like chemotherapies in the treatment of triple negative breast cancers, which needs further in vivo validation.

Abnormal alternative splicing has already been implicated in cancer progression such as cell proliferation, programmed cell death, metabolism, angiogenesis and metastasis [34, 35], rendering splicing an attractive therapeutic target for various types of malignancies[1]. For instance, inhibiting splicing was considered an effective approach to target multiple vulnerabilities of basal A type of TNBCs which reply on RNA splicing for survival [36]. Tumor cells have evolved abilities to hijack the RNA splicing machinery to reprogram gene expression towards their own advantages. It is likely that SNRPD1 over-representation leads to over-expression of genes promoting cell progression and down-regulation of those with cell cycle inhibitory roles due to altered RNA splicing.

We also examined the effect of SNRPD1 on other spliceosome complex proteins using SNRPE as an example. As a result, no visible variation on SNRPE expression was found by silencing *SNRPD1* (Additional file 2: Figure S1), suggestive of the independent role of SNRPD1 in breast cancer survival. This does not exclude the possible existence of a cross-talk between SNRPD1 and other ribosome binding proteins such as SF3B1, SF3B2, RPL5, ARCN1, EIF3B, RAN, COPB1, RPL14, VCP, HSPE1, SNRNP200, SARS, EEF2, RPL37, CCT3, KPNB1, RPL23 that have been reported essential for breast cancer survival besides SNRPD1 [14]. However, these are beyond the scope of this paper that focuses on core Sm proteins.

It is worth mentioning that we did not examine the functionalities of SNRPD1 on mRNA splicing which has already been documented [37]. Instead, our focus is laid on the tight association of SNRPD1 with cell cycle to expand our understandings on the multifaceted roles of *SNRPD1* and splicing. Splicesome inhibition has been proposed as an effective therapeutic approach for treating MYC-driven breast cancers [38]. Breast cancer cell lines we used in this study include MCF7, MDAMB231, MDAMB361 and HCC1937, which are all not MYC-driven as compared with the quasi-normal cell line MCF10A (Additional file 2: Figure S2). Thus, cell lines we used in this study may not be vulnerable to splicesome inhibitors as to silencing *SNRPD1*. On the other hand, none of the known splicesome inhibitors (including pladienolide B, E7107, FR901464, meayamycin, spliceostatin A, and sudemycines, isoginkgetin, herboxidine) was reported to target SNRPD1, whereas SF3b was identified as the common target of pladienolides and spliceostatin A [39, 40], SAP155 was that of herboxidine [41], and MMP9 was that of isoginkgetin [42]. Thus, splicesome inhibitors, though modulating the splicing process, may not affect *SNRPD1* expression and thus achieve similar effects. As one evidence here, silencing *SNRPD1* did not affect the VEGF/VEGFR axis (Additional file 2: Figure S3) that was reported to be modulated by spliceostatin A [43]. In addition, due to cytotoxicity, spliceosome inhibitors may cause cell cycle alterations under appropriate dosing [44] which, however, is not through targeting SNRPD1.

## Conclusions

We identified the novel association of *SNRPD1* with cell cycle progression in breast cancers, and therefore proposed *SNRPD1* as a novel target for breast cancer control through halting cell cycle progression at the G_0_/G_1_ phase. We are the first to link the role of *SNRPD1* with cell cycle progression and explain the distinct clinical outcomes of breast cancer subtypes using its differential expression. This study implicates the therapeutic potential of *SNRPD1* in breast cancer control that might be expanded to other types of cancers and warrants the combined use of doxorubicin and drugs targeting *SNRPD1* in treating triple negative breast cancers, which are subjected to experimental validations.

## Supplementary Information

**Additional file 1: Table S1.** Information on siRNAs purchased for knocking down *SNRPD1* in the study. **Table S2.** Information on the qPCR primers used in the experiment. **Table S3.** Information of 434 SRTNS from data analysis using protein MS data. **Table S.** Key cell cycle related Reactome pathways enriched by 434 SRTNS. The prediction was conducted using STRING. **Table S5.** Expansion of abbreviations in Fig. 2a.

**Additional file 2: Figure S1.** SNRPE protein expression in MCF7 and MDAMB231 cells on *SNRPD1* silencing. **Figure S2.** MYC gene expression in different breast cancer cell lines and quasi-normal breast cancer cells from (A) GSE12790 and (B) E-MTAB-181 datasets. MCF10A was the quasi-normal breast cancer cell line. The GSE12790 dataset [45, 46] and E-MTAB-181 [20] dataset were retrieved from GEO and ArrayExpress, respectively. The data was normalized using the robust multichip average (RMA) approach from the R package ‘affy’. **Figure S3.** VEGFR2 protein expression in MCF7 and MDAMB231 cells on *SNRPD1* silencing.

## Data Availability

All data used are publicly available.
