# Supplementary material for: SNRPD1 confers diagnostic and therapeutic values on breast cancers through cell cycle regulation
Source: Cancer Cell Int. 2021 Apr 20;21:229. doi: 10.1186/s12935-021-01932-w (PMC8059192; doi:10.1186/s12935-021-01932-w)
Supplement: Supplementary file 2 — Additional file 2: Figure S1. SNRPE protein expression in MCF7 and MDAMB231 cells on SNRPD1 silencing. Figure S2. MYC gene expression in different breast cancer cell lines and quasi-normal breast cancer cells from (A) GSE12790 and (B) E-MTAB-181 datasets. MCF10A was the quasi-normal breast cancer cell line. The GSE12790 dataset [45, 46] and E-MTAB-181 [20] dataset were retrieved from GEO and ArrayExpress, respectively. The data was normalized using the robust multichip average (RMA) approach from the R package ‘affy’. Figure S3. VEGFR2 protein expression in MCF7 and MDAMB231 cells on SNRPD1 silencing. [file 12935_2021_1932_MOESM2_ESM.pptx]

## Slide 1
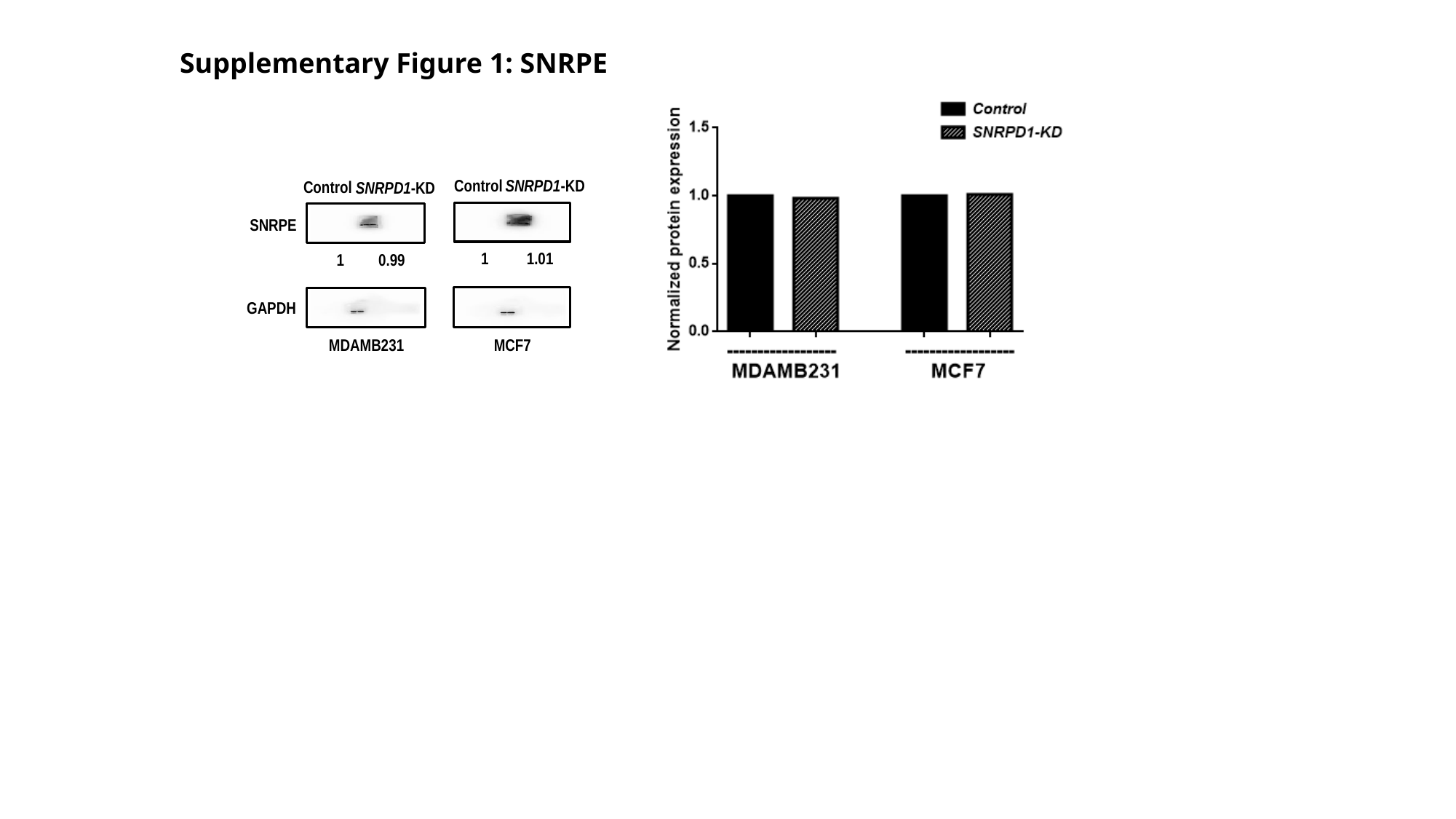

Supplementary Figure 1: SNRPE
SNRPD1-KD
Control
Control
SNRPD1-KD
SNRPE
1 1.01
1 0.99
GAPDH
MDAMB231
MCF7

## Slide 2
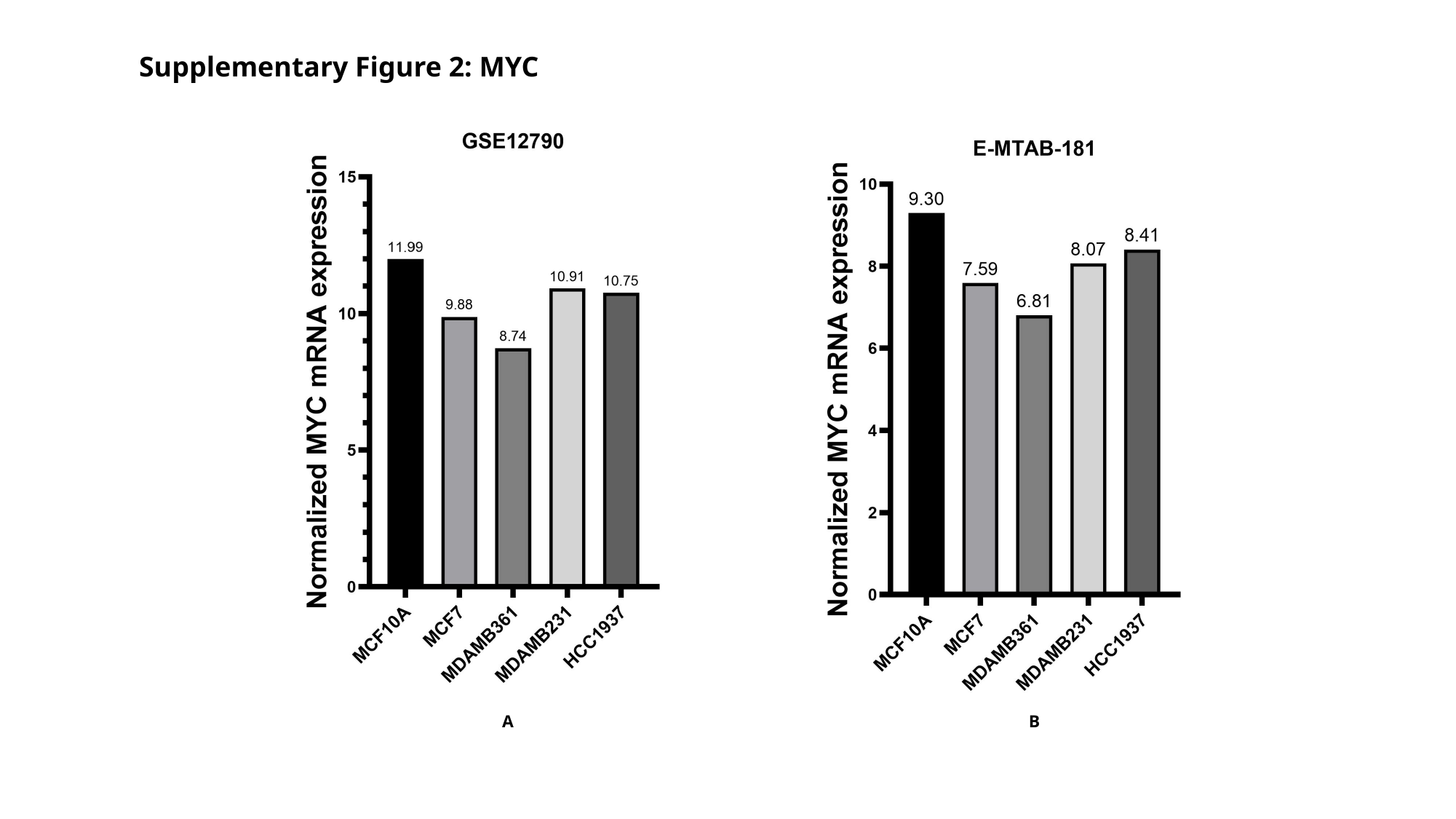

Supplementary Figure 2: MYC
B
A

## Slide 3
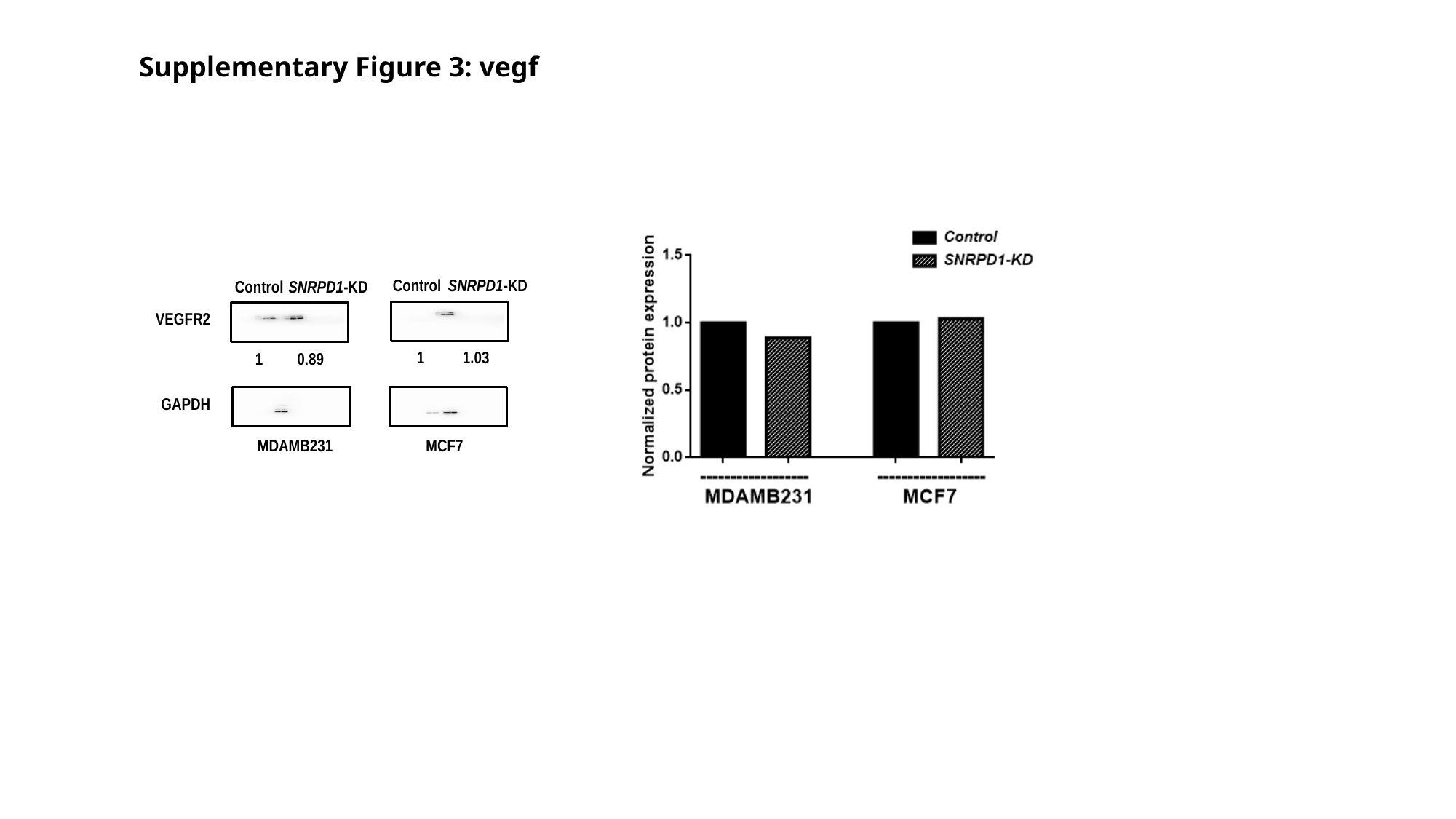

Supplementary Figure 3: vegf
SNRPD1-KD
Control
1 1.03
MCF7
Control
SNRPD1-KD
1 0.89
MDAMB231
VEGFR2
GAPDH
